# Deaf identities in a multicultural setting: The Ugandan context

**DOI:** 10.4102/ajod.v4i1.69

**Published:** 2015-05-26

**Authors:** Anthony Mugeere, Peter R. Atekyereza, Edward K. Kirumira, Staffan Hojer

**Affiliations:** 1 Department of Sociology and Anthropology, Makerere University, Uganda; 2 Department of Social Work, University of Gothenburg, Sweden

## Abstract

Often located far apart from each other, deaf and hearing impaired persons face a multiplicity of challenges that evolve around isolation, neglect and the deprivation of essential social services that affect their welfare and survival. Although it is evident that the number of persons born with or acquire hearing impairments in later stages of their lives is increasing in many developing countries, there is limited research on this population. The main objective of this article is to explore the identities and experiences of living as a person who is deaf in Uganda. Using data from semi-structured interviews with 42 deaf persons (aged 19–41) and three focus group discussions, the study findings show that beneath the more pragmatic identities documented in the United States and European discourses there is a matrix of ambiguous, often competing and manifold forms in Uganda that are not necessarily based on the deaf and deaf constructions. The results further show that the country's cultural, religious and ethnic diversity is more of a restraint than an enabler to the aspirations of the deaf community. The study concludes that researchers and policy makers need to be cognisant of the unique issues underlying deaf epistemologies whilst implementing policy and programme initiatives that directly affect them. The upper case ‘D’ in the term deaf is a convention that has been used since the early 1970s to connote a ‘socially constructed visual culture’ or a linguistic, social and cultural minority group who use sign language as primary means of communication and identify with the deaf community, whereas the lower case ‘d’ in deaf refers to ‘the audio logical condition of hearing impairment’. However, in this article the lower case has been used consistently.

## Introduction

There are different discourses on the concept of deaf identities^1^ ranging from whether or not it should be plural, to the interrogation of competing theoretical perspectives that account for their definitions and diversities (Bat-Chava 2000; Maxwell-McCaw, Leigh & Marcus 2000; Waqar, Atkin & Jones 2002; Leigh 2009; De Clerck 2010; Mcllroy & Storbeck 2011). Whereas deaf persons are widely considered a socially vulnerable group, some scholars have argued that contemporary deaf identities are crafted by balancing vulnerability and empowering forces (Breivik 2005b). According to Breivik, the question of vulnerability of being can be transformed into strength, especially when the deaf community arrives at a collective sense of belonging through sign language, deaf culture, socialisation and shared experiences (Hannah 2011; Hole 2007; Marieme 2013).

## Literature review

Discourses on deaf identities became prominent in the 1960s following the recognition of the American sign language and the growth of an international deaf community in the United States (Monaghan et al. 2003:28). The roles of deaf politics, ethnicity, gender and age in deaf identities have been examined in some studies. Waqar et al. (2002) discount notions of singular or primary identities (such as ‘deaf’ people or ‘Muslims’) in a study of Asian (mainly Pakistani Muslim) deaf young people and their parents in the United Kingdom. The authors describe Asian deaf young people's identifications as ‘multiple, complex and contingent’. In other words, they view claims to identity by their study population as a fusion of a host of socio-cultural factors that go beyond just having an hearing impairment and/or the use of sign language. Specifically, they attribute deaf identity development to a backdrop of deaf politics, ethnicity, religion, gender and age amongst other factors. Kusters (2009) examines the way deaf persons in ‘shared signing communities’^2^ (in Mexico, Bali, Israel and Ghana) participate in village life which is largely in the same ways as hearing people do – as members of social and linguistic groups. The author argues for more sustained fieldwork in studying Western deaf communities and shared signing communities to understand the ‘deaf-specific experiences and relationships in dynamic complex realities’ (Kusters 2009:7). Cline and Mahon (2010) also contend that deaf persons who share a common language such as the British sign language (BSL) find it easier to identify with other deaf people rather than with members of an ethnic or cultural community of similar origin to their own; a position strongly supported by Skelton and Valentine (2003) in their analysis of the role of BSL in young people's redefinition of their deaf identities.

In sub-Saharan Africa, research on deaf identities is quite limited. Lee (2012) examines the relationship between deaf people and mainstream society in Tanzania, concluding that there is no unified discourse on deaf identities in eastern Africa, with most work being conducted in South Africa (see, for example, Aarons & Akach 2002:134 ; Heap 2003). Nassozi and Donald (2003) explore several issues on deafness in the sub-continent but little emphasis is put on deaf identities. Schmaling (2000) assesses the significance of the *Sarkin Bebaye* [Chief of the Deaf], whose office is regarded as that of a representative of the deaf, paralleling the system of chiefs in the Hausa society of Nigeria. The issue of deaf identities was, however, none of his study themes. Similarly, other studies on the deaf in Africa over the last decade (Bisol 2008; Enwereji & Enwereji 2008; Groce *etal.* 2007) generally focus on other research themes such as HIV prevention and knowledge amongst the deaf, and health–related attitudes and behaviour, and differences in knowledge about HIV and AIDS. It is, therefore, evident that there is a paucity of empirical evidence on deaf identities in Africa. Whilst the numerous studies carried out elsewhere offer useful insights into this subject, they mainly explore issues of young deaf persons on a smaller scale and in less heterogeneous communities. Besides, they use mixed methods research approaches with no in-depth exploration of deaf persons’ identities and experiences. This partly presents a compelling case to conduct the Ugandan study.

## Multiculturalism in Uganda

Uganda, one of the smallest countries in eastern Africa, is also one of those with a complex cultural, ethnic and linguistic system in sub-Saharan Africa (Namyalo 2010). Its rich and diverse heritage and culture ‒ handed down in stories, folklore and songs from one generation to another ‒ are key pillars of the country's tourism industry and national identity. According to Gordon (2005), Uganda has 43 languages spoken by its estimated 34 million people (Uganda Bureau of Statistics 2012) broadly categorised into the Bantu, Sudanic, Eastern Nilotic and Western Nilotic ethnic groups. Such multilingualism and ethnic heterogeneity has facilitated the adoption and use of foreign languages such as Swahili and English, the latter being considered the official language for education and communication purposes (Namyalo 2010). The country's multicultural system is based on a closely-knit network of tribal and social groupings whose identities are shaped by varying norms, values, symbols and beliefs. Over the centuries, such a system has had a major impact on the construction of the identities of individuals and communities, including those of deaf persons.

In Uganda, deaf persons are officially categorised into the congenitally deaf, prelingually deaf, post lingually deaf and the hard of hearing (Douglas 2007; Lane 1975; Kathee 1998; Uganda National Association of the Deaf [UNAD] 2010). The congenitally deaf are those born deaf and never heard of any spoken word at all. They largely rely on the ‘Ugandan sign language’^3^ or gestures for communication and do not learn the spoken language of the surroundings—with a few exceptional cases. The prelingually deaf were born hearing but lost their sense under the age of five. Some of them cannot, therefore, ably speak the spoken language of the hearing community. The postlingually deaf were born hearing and lost their sense after learning the spoken language of the surroundings after the age of five. Some can, however, if provided with the necessary support (technological or otherwise), speak the language of the hearing community. On the other hand, the hearing impaired are those who can hear the spoken language to a certain extent but may take time to receive the message. Although there are isolated cases of some Ugandan deaf persons who use technological hearing aids amongst the above categories, none of such individuals was involved in this study.

### Aim and research questions

The main aim of this article is to explore the deaf identities and experiences of living with a hearing impairment in Uganda. The study, of which the article is an output, ^4^ was only designed to hear the ‘voices’ of the deaf individuals and no quantitative evidence of any aggregated data was provided. Some of the research questions that guided the study were: how do deaf persons understand their identities?; who do they interact with most?; what is the experience of living as a deaf person in a multicultural environment dominated by hearing people?

### Theoretical positioning

The theoretical paradigm within which this article is located is the social identity theory (Tajfel 1981) which posits that members of minority groups achieve positive social identity by attempting to gain access to the mainstream through individual mobility or working with other group members to bring about social change (Bat-Chava 2000). This perspective is adopted for its relevant assumptions which provide for the utilisation of a combination of both strategies to attain certain forms of identities. The idea that people are willing to see their group as better in some way than others—hence its ability to explain a wide range of social phenomena—also made the proposition of using the theoretical framework for this study attractive.

### Methodology: Design and setting

An exploratory-interpretive research design was used to uncover new ideas (Stebbins 2001) and incorporate various interpretive techniques that try to find the meaning, not the frequency, of occurring phenomena in the societal world (Cooper & Schindler 2003; Neuman 2003). It was based on unstructured interviews (in-depth conversations) with 42 deaf individuals (aged 19 to 41) and three focus group discussions (FGDs) – including one for female respondents – sampled from 10 districts of Uganda.

### Sampling procedure

The study areas and respondents were purposively sampled (Sarantakos 1997), a procedure that enabled the researcher to use his judgement to purposively choose only those areas and respondents who, in his opinion, were thought to be relevant to the research topic. The technique was also chosen to ensure maximum variation of study variables such as rural and urban settings, profession, age, marital status, level of education, additional forms of disabilities, values and beliefs, cause and age at which deafness occurred and family backgrounds until data saturation could be achieved. Purposive sampling was also considered appropriate because it was possible to generate the sample of deaf respondents (by text messages and mobilisation) through contacts at the national and district associations of deaf persons.

### Data collection

An interview guide in the form of a list of questions on the respondents’ lived experiences of being deaf was used. The sub-themes of the guide were perceptions and constructions of deaf identities, and experiences of deafness in a multicultural setting.

All the interviews were conducted in the Uganda sign language (USL) under the supervision of the lead author of this article with the help of experienced research assistants and USL certified interpreters between September 2012 and July 2013. Before starting any interview, the researchers fully explained the purpose of the study in the local language (with simultaneous USL translation) to the respondents and assured them of confidentiality, anonymity, voluntary participation and the right to withdraw from the proceedings at any stage of the interview. The lead author, who is not deaf but has since learnt the basics of the USL, also ensured that respondents who accepted to participate in the study signed consent forms to confirm that they willingly volunteered to take part in it and fully understood its objectives and scope. In cases where the respondents were illiterate and could not sign the consent forms, the lead author had to strike a compromise between the ethical codes and situational common sense (Mattila 2011) by explaining and empowering them to give consent by nominating one of their significant persons^5^ to sign on their behalf. During each of the one to two-hour interview sessions, one sign language interpreter used the ‘voice over’ system to interpret the interviewees’ USL signings whilst the second monitored the facial expressions and other reactions that could provide further insight into the subject of inquiry. Given the sensitive nature of the subject under investigation, it was important to monitor the respondents’ emotional reactions to the interview questions as they were used to provide further clues on what to probe for. Secondly, the facial expressions and other forms of reaction by the respondents were also used as an indicator of whether or not they had clearly understood the question. Thirdly, they were used to ascertain whether or not the respondents were freely giving their responses as many of them had never been involved in such a type of study interviews. The interviews were audio-recorded (with a few video recorded where USL was used), with the participants’ permission and written consent (signed in English).

### Data analysis

Audio recordings of the sign language interpreters and video recordings of both personal interviews and focus group discussions formed the data for this study (Wickenden et al. 2012). The video recordings were converted to DVDs using Adobe Premiere Pro CS44.0.1 video software (Mprah 2013). The transcription of the data from the DVDs was carried out in two steps, namely ‘partial’ transcription and full transcription. The first step (‘partial’ transcription) involved viewing the DVDs from all the focus groups to identify and transcribe into text format concerns that were raised by participants (Mprah 2013). These were, together with the ‘voiced over’ recordings transcribed to text format by certified USL interpreters who were used to develop the codes using Nvivo 10 qualitative analysis software (Gibbs 2012). The lead author organised the codes into themes, based on the specific objectives of the study. It was on this basis that the sub-themes (as presented in the results and discussion section of this article) and the analysis and interpretation of the study findings was carried out phrase by phrase (Miller 2005).

### Ethical and validity considerations

Fully aware of the limitations of qualitative research in which the researcher is often in direct contact with people (De Laine 2001), the lead author tried to avoid the ‘goodwill’ trap in which the interviewer has the role of a professional as well as sympathetic fellow being (Nasman & Eriksson 1994). Dissemination was carried out by the lead researcher in selected communities to provide feedback to the deaf individuals through workshops using videos with simultaneous USL interpretation. Further, to manage one of the most widespread criticism of qualitative studies, namely; the view that their results cannot be generalised to a wider population and the sample size and extent to which data saturation is reached is not quite convincing; rigour was attained by double-checking and, in some cases, focusing on how the analysis of the data evolves into a persuasive narrative‒a procedure described by Patton (1980) and as cited by Creswell and Miller (2000) as one where qualitative analysts return to their data ‘over and over again to see if the constructs, categories, explanations and interpretations make sense’.

## Results and discussion

Two major themes, namely, perceptions and construction of deaf identities in Uganda, and experiences of deafness in a multicultural environment, and eight sub-themes (Table 1) were identified to inform this article.

**TABLE 1:** Themes, sub-themes and key issues of the study.

Theme	Sub-theme	Key issues
Perceptions and construction of deaf identities	Culturally deaf identity	Positive attitude to deaf condition Resistance to deaf based discrimination Empowerment by advocacy groups
	Culturally hearing identity	Frustration and/or anger at being deaf Loneliness and withdrawal from the public Pretence to belonging to the hearing world
	Bicultural identity	Desire to belong to both deaf and hearing worlds Very active in NGO and other civil society work
Experiences of deafness in a multicultural environment	Culture	Unsuitable’ Uganda sign language gestures Difficulty in adopting some Uganda sign language alphabet to the local languages spoken in their localities.
	Religion	Muslim women forbidden from finger spelling certain Uganda sign language words Arabic literature incompatible with Uganda sign language
	Gender	Discrimination against female deaf persons Rejection by prospective hearing male suitors
	Family	Lack of social support Polygamy and extended family systems
	Linguistic diversity	Many dialects complicate use of Uganda sign language

It was on the basis of these themes that the findings of this article are presented and discussed.

### Sub-theme 1: Perceptions and construction of deaf identities in Uganda

Bat-Chava (2000) and Maxwell-McCaw *etal.* (2001) identified the salient indicators of deaf identities as personal and cultural identification, knowledge of deaf culture and preferences and involvement. It is on the basis of this criteria this study was conceptualised.

The overriding finding from the data is that the perceptions and construction of deaf identities by deaf persons in Uganda depend on the surroundings within which they are born, grow up and live. Generally, the study shows that most deaf persons have a positive attitude towards their condition. With a majority of them able to use USL, there is an overwhelming feeling that deafness is just a human experience. ‘I was born deaf but have never pitied myself at all. I have always believed that deafness is not a disability. It is a condition like any other’, remarked one respondent:

Another respondent: ‘I don’t know why I can’t talk but I am not angry with myself. I am married to a hearing wife, have three beautiful hearing daughters who are in good schools. I also have a car even if I hire someone to drive me around.

Another added: ‘There are times when I enjoy and dance to music played at parties. I look at the movements of those who hear to get the beats. My only problem is that I don’t hear the speeches made because there is no USL translation’.

A participant in a FGD commented: ‘I really hate those who discriminate against us because we are deaf. We are human beings like any other. Let the government build more technical training colleges for the deaf’.

Similarly, there was a related response: ‘My wife is not deaf but she is learning the USL. Normally, she understands my gestures and reads my lips quite well. But when she fails to get what I mean, I write it on a piece of paper. So I am happily married’.

The above responses typify what most researchers in deaf studies refer to as a culturally deaf identity. To such deaf persons, the extent of hearing loss is a non-issue in their lives. ‘I use the USL but it is still a language’, remarked one female focus group discussion participant. ‘I know there are many people who don’t understand it but it is the same with English. How many people can speak it?’, another asked rhetorically. They resist all forms of discrimination against them based on their condition and have been at the forefront of advocacy programmes to empower their community in various ways. One such programme has been the campaign for all local television stations to use captions and simultaneous USL translation during news bulletins and other broadcasts ‒ a move that has since been enforced by the Uganda Communications Commission (UCC). ‘These captions have really helped us feel that we are part of the general society. In fact, even some persons with no hearing impairments rely on these captions, so we are all together in this’, commented a deaf respondent.

However, the study's findings also show that there are deaf persons who are not at ease with their condition. Some of them expressed anger at why they should use sign language whilst the majority of their community members are hearing. ‘There are times when I think that God hates me’, one respondent said. Another commented: ‘I asked my parents several times why I can’t talk yet my brothers do but they gave me no answer … It is annoying’. Other respondents with similar identity traits said they preferred to be lonely most of the time to avoid being labelled ‘sick’. An FGD participant said that she sometimes chooses to pretend to be hearing to avoid being labelled deaf. ‘When I sit in a taxi, I wear headphones so that passengers who want to exit just tap my back instead of talking to me. They would assume that I am listening to music on my phone yet I am deaf’, she explained. Such respondents show a marked difference in attitude to deafness and would therefore wish that they could be ‘fixed’ medically, a view supported by proponents of the medical model of disability. ‘I wish there is a hospital in this country that could offer surgery to permanently heal my hearing impairment’, asserted a 36-year-old deaf male respondent. ‘I would sell whatever property is in my possession so that I can become a normal person’. Another 29-year-old hard of hearing male respondent, added: ‘I understand that the Europeans have machines that can heal deafness. But here in Uganda, there is none and that is disappointing for me … being born in such a developing country’. Under the Bat-Chava (2000) categorisation of deaf identities, such respondents would be regarded as culturally hearing.

This study also shows that there are deaf persons whose deaf identities can be categorised as ‘bicultural’. The concept of biculturalism encompasses the notion that an individual is able to gain competence within two cultures without having to choose one culture over the other (La Fromboise et al. 1993). These individuals not only spend much of their lives grappling with the contradictions of navigating between the different cultural groups but also interacting with both the hearing and fellow deaf persons. Most of their decisions are made basing on the desire to bridge the ‘gap’ between both hearing and deaf identities as observed by one respondent:

‘I had a hearing girlfriend but felt at some stage that we don’t communicate very well. She failed to learn USL and my gestures were becoming a problem especially when there was no light. Even some of her relatives were undermining me because of my disability so I now have a deaf woman and we are planning to marry soon’.

Although this study revealed evidence of heightened community sensitisation and advocacy for the deaf rights in Uganda, findings further show that the perceptions and construction of deaf identities is also influenced by the distance between them. A combination of physical barriers, poor infrastructure and high levels of poverty in most parts of the country makes it difficult for deaf persons to come into contact with one another. Despite the increasing use of text messaging and mobilisation by community workers amongst the community in the countryside, many are still not aware of other persons in their community who are deaf. ‘I am the only deaf person in this village so maybe that is my identity’, said one respondent. For such respondents the social and personal preferences they hold are, therefore, dictated by the fact that they must identify with the hearing community. They are forced to overcome all the barriers of their condition by making choices of friends or spouses from the only pool of hearing persons available in their communities. The development of the deaf identity features as identified by Bat-Chava (2000) and Maxwell-McCaw *etal.* (2001) would be dictated by the surrounding circumstances and not by choice or preference. Their knowledge of deaf culture and involvement in social clubs and other activities in the area would for instance be more a result of lack of knowledge of other persons than choice or preference. It would therefore be unrealistic for anyone to categorise such persons under any of the above deaf identities. This is also fully explained by the social identity theory that informs this study. As minority individuals, these ‘lonely’ persons seek social identity by working with (any) other group members to bring about social change in their lives. Regardless of whether or not he or she would wish to marry, he or she must find a marriage partner within the community, dominated by hearing persons. The idea that some persons are willing to see their group as better in some way than others, as argued by Bat-Chava (2000), therefore, becomes irrelevant. These findings concur with those of a study in Tanzanian where it was found that there is no unified discourse on deaf identity or a global deaf identity either (Lee 2012). With the exception of a few elites who have travelled internally, the deaf Tanzanians were found to have little awareness of deaf people outside their own communities.

Another key issue in the perception and construction of deaf identities in Uganda that emerged from the study was the role of civil society organisations. Although it is widely acknowledged that Non-Governmental Organisations (NGOs) have for the past two decades been at the forefront of mobilising and empowering deaf individuals countrywide, this study revealed that they are also shaping a form of deaf identity amongst their members. Despite their expressed sense of isolation from other deaf people, many respondents said that their membership of some NGOs that deal with issues related to the deaf community has in many ways fostered a special form of ‘bonding’ that has enabled them to attain personal and cultural identification, widen their knowledge of deaf culture and preferences and to become involved in many activities that they would never have dreamt of. One of the hallmarks of this form of bonding is the regular NGO meetings and other activities that include sensitisation seminars during which they also sign for allowances to attend, as explained by one FGD participant:

‘I used to feel so lonely out there. I used to think that I am useless until our NGO invited me for a seminar. They give us allowances to attend seminars which enable me to afford basic needs like food, medical care and mobile phones to ease our communication. We are also able to socialise, know other deaf persons from different parts of the county and avoid boredom’.

In many ways, this ‘NGO-centric’ form of deaf identity has helped galvanise and improve the living conditions of a section of the Ugandan deaf community. They regularly meet to share experiences, learn vocational skills and promote the USL. Some of them indicated that they had formed small savings and credit organisations to boost their economic status and sports clubs for recreation purposes. The Ugandan NGOs have therefore unconsciously given some persons theopportunity to understand who they are and to define their characteristics, like in Tanzania where the deaf in urban areas are unified by the characteristic of being ‘deaf’ and held together by a range of social networks produced and reproduced through face-to-face gatherings during meetings of deaf organisations and clubs, participation in sports formats (such as Olympics and basketball championships) and beauty pageants (Lee 2012). In a way, this is a form of deaf identity even if it is less ideologically oriented and more pragmatic than those documented in American and Western discourses.

Furthermore, those who sign for the allowances indicated that they do so on account of being deaf whose plight is being responded to by the hearing world (through donations and other forms of support). This is against the backdrop of the widely held view amongst researchers that the practice of signing for seminar allowances is not unique to deaf persons. Lee (2012) also notes that Tanzanian NGOs dealing with the deaf population – whether national or regional – only serve as ‘sites of transmissions of internal discourses of deaf culture’ to the most elite of such persons. Branson and Miller (2002) also discuss the same scenario in ‘Damned for their difference’ thesis on identity politics.

### Sub-theme 2: Experiences of deafness in a multicultural environment

The study results show that the experiences of deafness in Uganda are greatly shaped by the country's religious, cultural, gender, family and linguistic diversity. In some parts of Uganda, female Muslim deaf persons are discouraged from using particular USL finger spellings and gestures by their significant persons and religious leaders whilst communicating with men, except to their husbands or other men to whom they are closely related. In most cases, some Islamic clerics preach that a Muslim woman who uses certain forms of finger spellings and gestures to communicate to a man risks being misunderstood by the man or even society (which is dominated by persons with no hearing impairment) as an indicator that the woman is initiating an intimate relationship, which is in itself a taboo. Other Muslim faithful believe that there should be no form of contact or communication whatsoever between a Muslim woman and a man to whom she is not closely related or married too. For such believers, a ‘true’ Muslim woman is not only judged by the way she ‘veils’ her body and adheres to the pillars of her faith but also the distance she keeps from men (whom she is not married or related to), including refraining from using ‘obscene’ USL finger spelling and gestures to them.

Although this is aimed to promote abstinence and faithfulness amongst Muslim women, it restricts the way they talk to and interact with men, for official and social purposes. The results further show that there are some cultures and customs in central Uganda that consider a few of the USL finger spellings and gestures ‘obscene’ and unsuitable for use in public. For such communities, the use of certain gestures and other forms of signing point to insinuations of sexist expressions whose use in public is forbidden. Therefore, anyone (including deaf persons) who uses such expressions is considered a ‘spoilt’ person who publicly expresses love feelings, a form of behaviour akin to that of prostitutes seeking to attract clients. Consequently, many deaf persons – especially women – fear to use USL finger spellings and gestures which are regarded as ‘obscene’ by society. In effect, this limits not only their freedom of expression but also the use of USL.

Whilst this study did not explore social support systems for the deaf persons per se, it emerged that families, friends and neighbours play an important role in the lives of this group. Several deaf persons indicated that they were born and raised by their parents on equal footing with their hearing siblings. They received good child care, were taught USL and had attended schools which were accessible to them. Some of them currently hold well paying jobs in the private and public sectors with varying levels of influence in national and international arenas. However, there are those for whom decades of insurgency, disease and ignorance brought untold suffering over the years. These include the orphans of war and the HIV and AIDS pandemic, some of whom were forced to become household heads before their teenage years to look after their hearing siblings. With no adequate formal support structures in the country, their experiences are daunting, as observed by an FGD participant:

‘For me, life has been hell on earth. My parents died of AIDS and I became the parent of my brothers and sisters when I was just 13. The liberation war of the 1980s also made me a war veteran. We have a small piece of land to cultivate, so there is always little food at home. These terrible events are even worse than my deaf condition’.

From the above quote, it is evident that in the face of widespread poverty, this socially vulnerable group is also one of those hit hardest by disease, ignorance, malnutrition and the general lack of the means of production. It also points to the overall gaps in the identification and intervention into the socio-economic problems affecting them. Respondents also described additional forms of disabilities (such as physical disability and blindness), polygamy and wider family networks as compounding factors to their plight, such as this 26-year-old deaf male's narrative: ‘I am deaf but also walk on one leg (with the aid of a stick) because I was crippled by polio in my early years. I also know of another deaf person in the neighbouring district who is blind. It is double tragedy for many of us’.

Another 30-year-old female respondent explained:

‘Some of us are married to men who have other wives without any hearing impairment. My husband is one of them and my co-wives insult me that I am just a deaf woman who came to produce deaf children in this family … Even some of his relatives feel bad that he chose to marry me, a deaf woman. They do not talk to me even when we meet for family functions such as weddings and funerals’.

Interwoven into this matrix of variables are the gender perceptions and constructions which largely affect the female deaf persons. Whereas there were two cases of female respondents who were married to deaf spouses, the study results show that very few hearing men would be willing to marry a deaf woman. This often creates relationship complexities at household level as the majority of eligible deaf women remain either unmarried or marry hearing men that they fail to bond with. ‘The hearing men think we cannot fulfil our marital obligations because of our hearing impairment’, observed one 27-year-old female deaf respondent. ‘They also probably fear that we will give birth to deaf children and this further discourages them from marrying us’.

These findings resonate with those of the Tanzanian study which found that deaf women have limited recourse in traditional community structures, live away from their biological families and rely on members of their deaf networks for support as the long-standing traditional networks – such as parents and community members – are often inaccessible to them (Lee 2012). Nassozi and Donald (2003) also explore the ‘triple discrimination’ faced by deaf women in other sub-Saharan African countries because of deafness, gender and poverty.

*These* *Being Deaf and being other things* (Waqar et al. 2002) findings not only further complicate the deaf identities but also underlie the living conditions of the deaf community in Uganda. In some districts, religion is not just faith, but a symbol of identity for believers. Membership to a particular religious group means that one has to acquire some identity features that characterise its followers, such as language and accent. For the deaf persons this is, however, impossible and therefore a limitation on the way they espouse their beliefs. The proliferation of Pentecostal churches and other groups in the country over the last 20 years has further complicated the situation. Whereas some deaf persons, for instance, attend special church services organised for them in the urban areas, those in the rural areas find it difficult to identify with the churches and mosques where their special needs are not catered for. The restoration of the Buganda kingdom and its cultural ruler, the Kabaka, and other kingdoms in 1993 renewed the significance of cultural values, beliefs and customs amongst those endeared to them. Although many deaf persons would be more willing to identify themselves with the main religious groups, tribes or cultures in the country, they undergo varying forms of cultural socialisation and ‘speak’ multiple dialects. Besides, some cultures still regard deafness as a curse to the family, clan or local community. Others are isolated, stigmatised and taunted using some derogatory vernacular words such as *kasirus, zontos* or *bubus* [idiots or imbeciles]. In some areas, the illiterate deaf persons rely on ‘home-made’ gestures and signs for communication which is a common feature of co-segregation and ‘Deaf-specific experiences’ in village sign languages (Zeshan 2005:560). Even if a few of them are integrated in their local communities by offering them odd jobs such as working as foremen at filling stations and digging pit latrines in people's homes, their ambitions are greatly constrained by the lack of the ‘empowering aspects of being Sign Language users’ (Breivik 2005a:18).

These findings show that the four stage phase of deaf identification described by Ohna (2004) partially applies to Uganda. Whilst it would be harsh to conclude that such deaf persons are ‘alienated’ or ‘taken for granted’, there are indicators that they are ‘Deaf in their own way’ (Breivik 2005a:10). For instance, deaf Muslim men who cannot speak Arabic (the recommended language for communication in Islam) attach greater significance to wearing the *kanzu* [tunic] than their hearing counterparts in defining their religious identity. Similarly, those who cannot ‘speak’ USL tend to complement their use of gestures by being obedient and the most result-oriented workforce in the informal sector where their services are valued. Lastly, some deaf Muslims also face challenges using the USL alphabet to finger spell words from their holy book, the Qur’an, the alphabet of which is based on the Arabic language. With the exception of the minority who attend Islamic schools where they learn signing in Arabic, the rest find it difficult or even impossible to follow the writings in the Qur’an and other Islamic literature. This also aligns with the theoretical orientation of this study in a sense that group affiliation has not only been internalised psychologically to describe the subjective self (Tajfel 1981) but is also used to define the categories of knowledge and of ‘reality’ created and are the products of social and symbolic relationships and interactions, all within the given temporal and spatial boundaries of a cultural context (Hacking 1999). In other words, whatever the deaf persons may individually espouse, is contingent on community values and perceptions which, in turn, affect the way they define themselves. In summary, the multicultural environment in Uganda is more constraining than enabling to the lives of the deaf community, akin to findings in Asia where a ‘hybridity’ of identity emerged as young deaf persons found it difficult to become full members of their religious and ethnic communities (Waqar et al. 2002).

### Implications

The findings in this article are expected to contribute to the theoretical discourse on deaf identities in developing countries. They also have practical implications on informing policy and programme design and implementation for the deaf community in most sub-Saharan African countries.

## Conclusion

This article examined the perceptions and construction of deaf identities and experiences of living as deaf in Uganda. The study findings showed that in addition to the deaf identities that have for long dominated discourses on this subject, the Ugandan setting provides for other factors that are not necessarily enshrined in the deafness constructions. This is clearly linked to the country's cultural, religious and ethnic diversity. That identities are situational and flexible is a truism (Hall 1992; Westwood & Rottansi 1994). It is therefore prudent for researchers to embrace these diversities in order to understand the lifestyles and social behaviour of this socially vulnerable group. Whereas some have posited that accessing an emic perspective without a shared formal language is definitely possible (Lee 2012), it is easier said than done whilst dealing with this ‘silent’ population.

## Footnotes

1 Identities, the plural form of identity is used in this article to refer to ‘a person's understanding of who they are and, of their fundamental defining characteristics as a human being’ (Taylor 1994).

2 ‘Shared signing communities’ is used in this article to refer to the ‘pervasive use of signing by both the hearing and deaf’ (Kisch 2008).

3 ‘Uganda sign language’ has been placed in quotation marks because Uganda is one of the countries where sign language is still in its formative years. Although it was formally recognised by government in the mid-1990s and there is a fully fledged Deaf Studies Programme at Kyambogo University, it does not have official status in schools. Even when it is used in classrooms, the sign language skills of the teachers are often not adequate because of insufficient training (UNAD 2010).

4 This article is an output of a larger study aimed to investigate the perceptions of meaning and response to HIV and AIDS information by deaf persons in Uganda. Its first specific objective (theme) was to explore the deaf identities and experiences of deafness in a multicultural environment.

5 The concept ‘significant persons’ in this study is a modification of Charles Horton Cooley's (1864–1929) definition of ‘significant others’ as used by Ritzer and Goodman (2004) to refer to people in our lives whose opinions matter to us and who are in a position to influence the way we think about things, especially about ourselves. This is contained in Cooley's theory of socialisation in which he argues that individuals develop their self-images through their interactions with significant others. Such persons include caregivers (such as family, friends and neighbours), sign language interpreters, religious and opinion leaders.
